# Carboxymethyl Dextran-Based Biosensor for Simultaneous Determination of IDO-1 and IFN-Gamma in Biological Material

**DOI:** 10.3390/bios15070444

**Published:** 2025-07-10

**Authors:** Zuzanna Zielinska, Anna Sankiewicz, Natalia Kalinowska, Beata Zelazowska-Rutkowska, Tomasz Guszcz, Leszek Ambroziak, Miroslaw Kondratiuk, Ewa Gorodkiewicz

**Affiliations:** 1 Bioanalysis Laboratory, Doctoral School of University of Bialystok, Faculty of Chemistry, University of Bialystok, Ciolkowskiego 1K, 15-245 Bialystok, Poland; 2 Bioanalysis Laboratory, Faculty of Chemistry, University of Bialystok, Ciolkowskiego 1K, 15-245 Bialystok, Poland; ania@uwb.edu.pl (A.S.); natala.2132@gmail.com (N.K.); 3 Department of Pediatric Laboratory Diagnostics, Medical University of Bialystok, Waszyngtona 17, 15-274 Bialystok, Poland; zelazowskab@wp.pl; 4 Department of Urology, Hospital of Ministry of Interior and Administration in Bialystok, Fabryczna 27, 15-471 Bialystok, Poland; tomasz.guszcz@o2.pl; 5 Department of Automation of Manufacturing Processes, Faculty of Mechanical Engineering, Bialystok University of Technology, Wiejska 45C, 15-351 Bialystok, Poland; l.ambroziak@pb.edu.pl (L.A.); m.kondratiuk@pb.edu.pl (M.K.)

**Keywords:** IDO-1, IFN-gamma, SPRi, bladder cancer

## Abstract

Indoleamine 2,3-dioxygenase 1 (IDO-1) and interferon-gamma (IFN-γ) are proteins that play a significant role in inflammatory conditions and tumor development. The detection of IDO1 and IFN-γ is crucial for understanding their interplay in immune responses. This study introduced a novel method for the simultaneous quantitative determination of IDO-1 and IFN-γ in different biological samples/materials. The method is based on an optical biosensor, with surface plasmon resonance detection carried out by the imaging version of the sensor (SPRi). Biotinylated antibodies immobilized on the surfaces of the linker and carboxymethylated dextran served as the recognition elements for the developed biosensor. Relevant studies were conducted to optimize the activities of the biosensor by employing appropriate reagent concentrations. Validation was performed for each protein separately; low detection and quantification limits were obtained (for IDO-1 LOD = 0.27 ng/mL, LOQ = 0.81 ng/mL; for IFN-γ LOD = 1.76 pg/mL and LOQ = 5.29 pg/mL). The sensor operating ranges were 0.001–10 ng/mL for IDO-1 and 0.1–1000 pg/mL for IFN-γ. The constructed biosensor demonstrated its sensitivity and precision when the appropriate analytical parameters were determined, based on the proposed method. It can also selectively capture IDO-1 and IFN-γ from a large sample matrix. The biosensor efficiency was confirmed by the determination of IDO-1 and IFN-γ in simultaneous measurements of the plasma and urine samples of patients diagnosed with bladder cancer and the control group. The outcomes were compared to those obtained using a certified ELISA test, demonstrating convergence between the two methodologies. The preliminary findings demonstrate the biosensor’s efficacy and suitability for comprehensive analyses of the examined biological samples.

## 1. Introduction

IDO-1 belongs to the family of enzymes known as intracellular heme dioxygenases, which initiate tryptophan catabolism along the kynurenine pathway (KP). The IDO-1 enzyme plays a key role in the oxidative metabolism of tryptophan, which is why it is considered to be one of the main antioxidant factors inside cells [1]. A total of 85% of tryptophan is converted by the indoleamine-2,3-dioxygenase one enzyme (IDO-1). Bioactive metabolites generated during tryptophan degradation assume pivotal immunoregulatory roles within the organism. IDO is primarily mediated by interferon-gamma (IFN-γ), underscoring its crucial role in the immune response [2,3].

IDO-1 is a 45 kDa monomeric protein, consisting of 403 amino acids and composed of two distinct alpha-helical domains of different sizes, with a heme prosthetic group between them [4,5]. It is coded by the INDO gene, which is located on chromosome 8p12 in humans, and is regulated by IFN-γ responsive elements [3]. Researchers have confirmed the critical role of IDO-1 in various biological processes, including those involving infectious viruses and bacterial diseases, transplantation, and cancers [1,6,7,8,9]. This enzyme plays a crucial role in mitigating inflammation and facilitating tissue regeneration. IDO-1 inhibits the activity of T lymphocytes and natural killer (NK) cells, while stimulating T-reg lymphocyte production and angiogenesis [10].

Interferon-gamma is a proinflammatory cytokine, produced primarily by T lymphocyte cells and NK cells in response to immunological or inflammatory stimuli. IFN-γ belongs to the glycoprotein family. It is a homodimer, with a mass of 34 KDa and a variable size and charge, due to cleavage of the carboxyl terminus and a variable degree of glycosylation. IFN-γ, encoded by the IFNG gene that is located on chromosome 12, is a polypeptide consisting of 146 amino acids, arranged in antiparallel chains [11]. It exerts a pleiotropic influence on the immune system, enhancing the cytotoxic activity of immunocompetent cells, such as T lymphocytes and NK cells, upregulating the expression of MHC molecules, activating macrophages, and promoting phagocytosis, as well as stimulating the production of various other cytokines [12].

The interaction of IDO with IFN-γ was first studied by Werner et al. They observed that IFN-γ-stimulated macrophages could degrade tryptophan, the main target of IDO-1 [13]. IFN-γ, via the activation of the JAK/STAT1 (Janus kinase/Signal Transducer and Activator of Transcription 1) signaling pathway, increases IRF1 (interferon regulatory factor 1) expression and affects IDO-1 transcription [14]. Both proteins are critically involved in the pathogenesis of numerous diseases, particularly malignant neoplasms. The complex and dynamically evolving tumor microenvironment is a critical factor in tumor progression. IDO-1 levels are elevated in the tumor microenvironment and are strongly associated with its malignancy [9,10,14]. Research indicates that IFN-γ plays a pivotal role in tumor immunosurveillance, contributing to the host’s defense mechanisms against cancer [11,15]. In regard to inflammatory conditions and tumor development, the detection of IDO1 and IFN-γ is crucial for understanding their interplay in immune responses. The upregulation of IDO1 by IFN-γ can suppress immune responses. Tumors use the relationship between these proteins to favor their immune escape. The simultaneous determination of these two proteins may be helpful in the prognosis and selection of the appropriate treatment. The IFN-γ-IDO1 pathway and its correlation were analyzed in cervical cancer [16] and in systemic lupus erythematosus [17]. In addition, a combination of IFN-γ treatment with IDO-1 inhibitors is a promising new immunotherapeutic strategy in cancer treatment [18].

The quantification of IDO-1 and IFN-γ can be helpful in the diagnosis of cancers and also in monitoring their treatment. IDO-1 activity is most often determined through the quantitative measurement of the produced kynurenine or by selecting the ratio of kynurenine to tryptophan concentrations [19]. ELISA tests [20,21,22] and the immunofluorescence [23] method were used to determine the level of IDO-1 in blood. An “on–off” SERS sensor, triggered by an enzyme, was also developed to detect IDO-1 in the presence or absence of colon cancer [24].

The IFN-γ concentration is mainly determined using immunochemical methods, such as the ELISA immunoenzymatic test or the ELISPOT method [25]. For the determination of IFN-γ in body fluids, biosensors, such as an electrochemical immunosensor [26], a quartz crystal microbalance (QCM) sensor [27], a liquid crystal (LC)-based aptasensor [28], and an electrochemical aptasensor with a bifunctionalized conducting polymer nanobioconjugate [29], have been described. Among the detection techniques used by biosensors, optical techniques, such as surface plasmon resonance (SPR), have received particular attention in the literature. The combination of biosensing with SPR can provide a route for conducting biomolecular detections in a simple, fast, and real-time manner. It also offers label-free quantification. SPR is a technique used to study the kinetics of bonds between biomolecules. This technique was used to determine the interaction between IDO-1 and Kushenol E [30]. SPR biosensors have also been developed to detect INF-γ [21,22,23,24,25,26,27,28,29,30,31,32,33].

Two main types of plasmonic models are used in SPR biosensors: propagating surface plasmons (PSPs) or so-called localized surface plasmons (LSPs) [34]. PSPs are oscillations of conducting electrons near the surface of the metal. Metal can occur in the form of continuous layers or strips. LSPs are supported by metal nanoparticles. One version of the SPR technique is SPR imaging (SPRi), which enables increases to the throughput and provides the necessary spatial resolution for observing molecular interactions. In a typical SPRi system, a polarized beam of light is used as the excitation light at a fixed angle, and the spatial distribution of particles can be detected by tracking the changes in the intensity of the reflected light recorded by a CCD camera [35]. SPRi biosensors enable a quantitative approach to be taken to the analysis of biomolecular interactions. They detect refractive index changes near the surface related to analyte mass information. Using an appropriate matrix on the biosensor surface allows for the simultaneous determination of analytes in many different samples. Such matrix SPRi biosensors are sensors with a polymer film surface that allows for the simultaneous measurement of multiple samples, causing the separation of bioreceptor substances. This allows for the simultaneous study of many interactions occurring on the immobilized surface and, consequently, for determining various biomolecules with diagnostic potential in biological fluids [36,37,38]. In recent years, the detection of viral and bacterial pathogens using SPRi sensors has also attracted interest [34,39].

This article presents a new method, based on an SPRi matrix biosensor, for the simultaneous determination of IDO-1 and INF-γ in biological samples. The method is based on using specific antibodies as elements that capture a given analyte. The analytical parameters of biosensors were characterized in detail and applied to the IDO-1 and INF-γ analysis of clinical samples, exhibiting good reliability, repeatability, and reproducibility. So far, the determination of IDO-1 has mainly involved determining its activity; however, the method developed in this study enables the determination of the concentration of both IDO-1 and INF-γ and the use of the results to study their mutual correlations.

## 2. Experimental Process

### 2.1. Materials and Reagents

The following reagents were used for the construction of the biosensor and the validation of the analytical method: 3-mercapto-1-propanol, carboxymethyl dextran, 1-ethyl-3-(3-dimethylaminopropyl)carbodiimide hydrochloride (EDC), 1-hydroxy-2,5-pyridinedione (NHS), epichlorohydrin, streptavidin, ethanolamine, sodium hydroxide (NaOH), polyoxyethylene sorbitan monolaurate (Tween-20), 2-[4-(2-hydroxyethyl)-1-piperazinyl]ethanol (HEPES), bromoacetic acid, diethylene glycol dimethyl ether, bovine serum albumin (BSA), and sodium lauryl sulfate (SDS), all obtained from Merck, Darmstadt, Germany. The following were also used: the recombinant monoclonal mouse anti-IDO-1 antibody, the recombinant monoclonal mouse anti-IFN-γ antibody, the recombinant human IDO-1 protein, and the recombinant human IFN-γ protein, obtained from R&D Systems, Minneapolis, MN, USA, as well as phosphate-buffered saline (PBS) (Biomed, Lublin, Poland) and absolute ethanol (PolAura, Morąg, Poland).

The composition of the basic buffers used: HEPES–EDTA–P20 solution 500 mL (10 mM HEPES, 150 mM NaCl, 3 mM EDTA, 0.05% Tween-20), pH = 7.40; PBS–EDTA–P20 solution (PBS + 0.05% Tween-20 + 3 mM EDTA), pH = 7.40.

### 2.2. SPRi Apparatus

**Figure 1:**
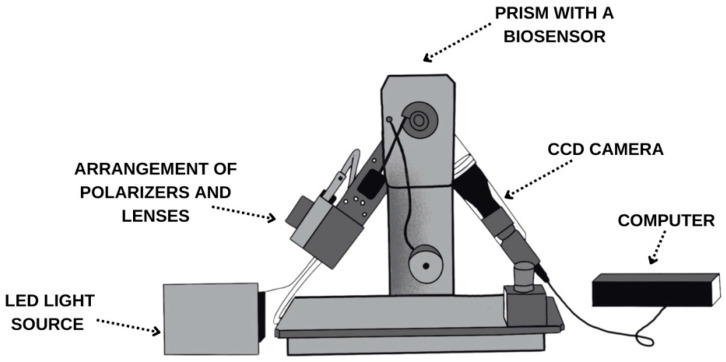
Schematic diagram of the SPRi device, with its component parts.

The SPRi device, constructed in the Bioanalysis Laboratory, Faculty of Chemistry, University of Białystok, was used for the research. Its components are shown in Figure 1. The device has movable arms that set the appropriate angle at which the measurement is performed. Numerical data acquisition is enabled by directing the light from an LED source through a system of polarizers and lenses, followed by its reflection on the surface of a gold sensor chip. The reflected light is then captured by a CCD camera, interfaced with a computer for data processing. The data is initially recorded as an image, and then mathematical operations are performed to transform the recorded images into numerical data.

### 2.3. Biological Material

This study involved the analysis of 27 plasma samples that were obtained from patients diagnosed with bladder cancer, along with 3 control plasma samples from individuals with prostatitis. Additionally, 26 urine samples from bladder cancer patients and 4 control urine samples from prostatitis patients were examined. The research protocol received approval from the Bioethics Committee at the Medical University of Bialystok (approval number APK.002.307.2023, dated 22 June 2023). Informed consent was obtained from all the subjects involved in the study.

In regard to the biosensor validation process, two samples from patients with brain glioma were also used, namely a blood serum sample and a tissue homogenate sample. The samples were sourced from the Biobank at the Medical University of Bialystok, and the requisite consent to conduct the study was obtained from the pertinent bioethics committee (approval R-I-002/600/2019—19 December 2019 and approval APK.002.171.2021—25 March 2021)

### 2.4. Construction of the Biosensor

#### 2.4.1. Receptor Layer Preparation

**Figure 2:**
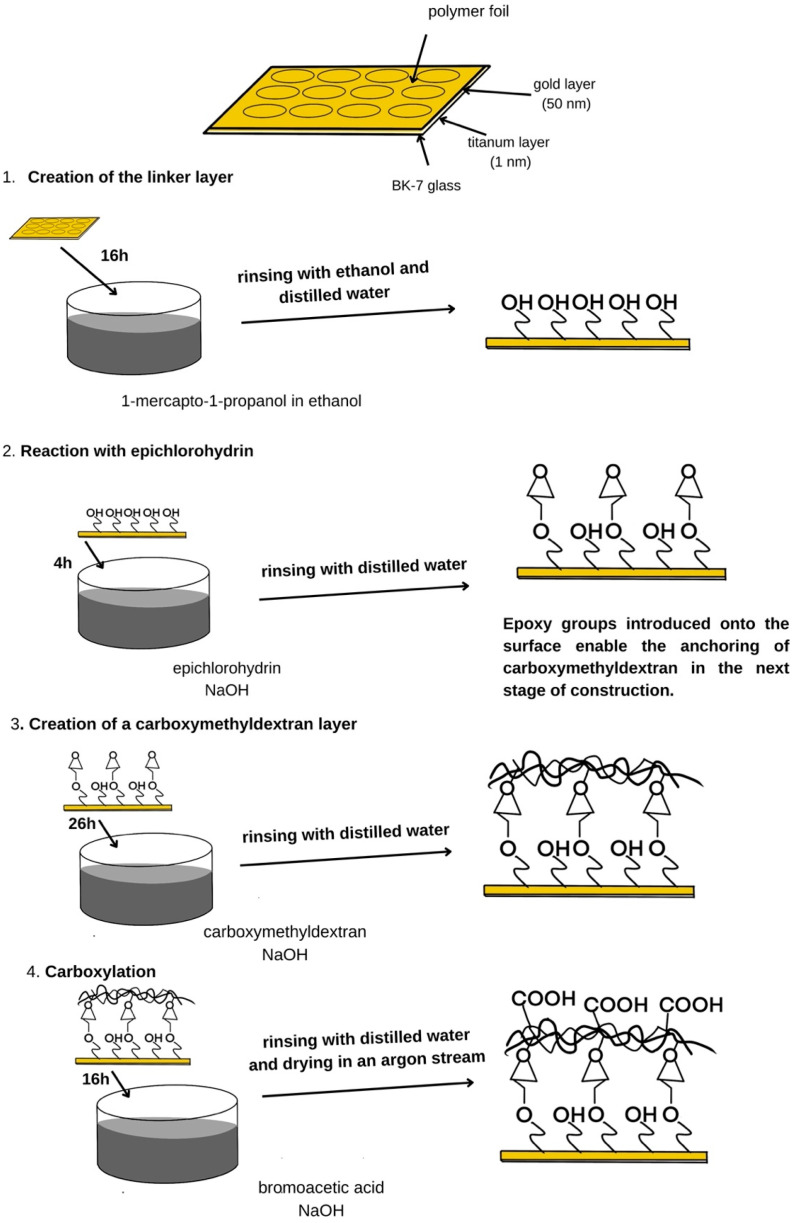
Gold layer preparation steps include the formation of a linker layer, reaction with epichlorohydrin, the formation of a carboxymethyl dextran layer, and a carboxylation reaction.

The initial step involves the formation of a linker layer on the biosensor surface using 3-mercapto-1-propanol. The plate with the gold layer was rinsed with absolute ethanol, dried in a stream of argon, and then immersed in a 1 mM alcoholic solution of 3-mercapto-1-propanol for about 16 h. Subsequently, the chip was rinsed once more with alcohol, followed by deionized water. The linker layer obtained through this procedure was subjected to a reaction with epichlorohydrin. The chip was immersed for 4 h in a mixture of epichlorohydrin (0.6 M) and NaOH (0.4 M), prepared with a volume ratio of 1:1. Then, the chip was rinsed with deionized water. A solution of carboxymethyl dextran at a concentration of 5% in 0.4 M of NaOH was prepared. A 5% solution of carboxymethyl dextran in 0.4 M of NaOH was prepared, and the gold-coated plate was immersed in this solution for 26 h. Following incubation, the plate was rinsed three times with deionized water. The subsequent step involved carboxylation, carried out by reacting the immobilized carboxymethyl dextran with bromoacetic acid. A solution of bromoacetic acid with a concentration of 1 M in 2 M of NaOH was prepared. The chip was immersed in the reaction mixture for 16 h, after which it was rinsed three times with deionized water and dried under a stream of argon. At this stage, a polymer film was also applied to isolate the measurement areas on the sensor surface. The complete procedure for preparing the gold-coated plate is illustrated in Figure 2.

**Figure 3:**
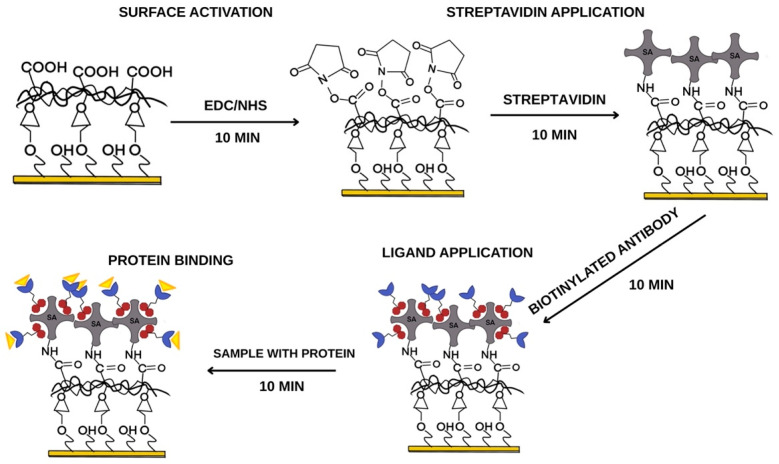
Preparation of the ligand–antibody layer.

The carboxyl groups formed during the reaction should be activated to create the appropriate amino ester group, enabling the bonding of subsequent layers. EDC (0.8 M) and NHS (0.2 M) solutions were mixed in a 1:1 ratio and applied to the active sites of the biosensor. A graph of the preparation of the ligand layer is shown in Figure 3. After 10 min, the excess solution was removed, and the sites were washed once with the basic buffer HEPES–EDTA–P20, pH = 7.40. A streptavidin solution (0.5 mg/mL) was introduced onto the sensor surface for 10 min. After the required time had elapsed, the excess solution was sucked off and rinsed once with the basic buffer (HEPES–EDTA–P20). Streptavidin forms a covalent amide bond with the carboxymethyl dextran groups, which are activated with EDC and NHS. To deactivate the groups not bound to streptavidin, an ethanolamine solution (1 M) was applied to the surface of the measurement sites for 10 min. After the required time had elapsed, the excess solution was removed and the sites were rinsed with the basic buffer. The last stage of construction involved the use of biotinylated antibodies specific for IDO-1 and IFN-γ. Biotinylated antibodies can form a biotin–streptavidin complex. The antibodies were applied to the measurement sites for 10 min; then, the excess solution was removed from the surface and rinsed once with the basic buffer. Biotinylated antibodies bind to all nine measurement sites where a carboxymethyl dextran layer has been formed, and streptavidin can covalently link the amino groups to the carboxyl groups of the carboxymethyl dextran.

#### 2.4.2. SPRi Measurement

The fabricated sensor with the immobilized receptor layer was mounted onto the prism of the SPRi system using immersion oil. The instrument’s adjustable arms enabled precise angle selection and the acquisition of a series of images across a defined angular range corresponding to the receptor layer. In the subsequent step, the samples were introduced into the measurement cell and incubated for 10 min to allow for the interaction between the immobilized antibodies and the target protein. Excess solution was removed after the required time had elapsed, and the active sites were rinsed with the basic buffer. A series of photos was retaken of the analyte layer. The optimal angle was selected based on the most significant signal difference between the ligand and the analyte and remained constant until the end of the analysis. The analytical signal, which is desired, is the difference in the intensity of the reflected light before and after the interaction with the analyte.

## 3. Optimization of Method Conditions

### 3.1. Selecting the Appropriate Streptavidin Concentration

**Figure 4:**
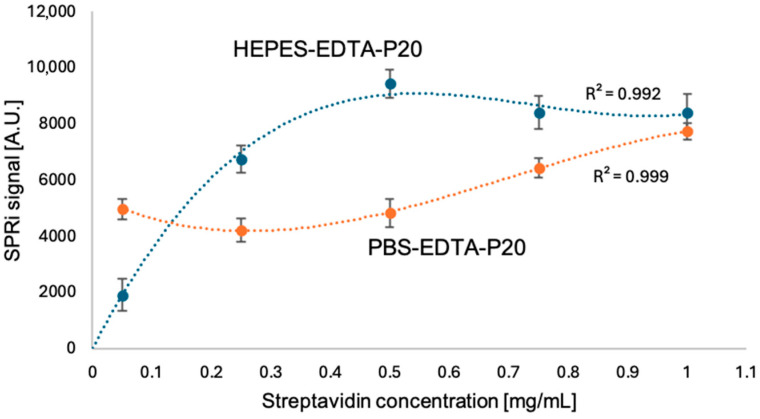
Dependence of SPRi signal on streptavidin concentration. Test performed in HEPES-EDTA-P20 basic buffer, pH = 7.40, and PBS-EDTA-P20 basic buffer, pH = 7.40.

Streptavidin solutions from 0.05 to 1.00 mg/mL were prepared, and the SPRi signal was measured. For this purpose, the SPRi signal of the carboxymethyl dextran layer was measured, then streptavidin was immobilized on it, and another SPRi signal measurement was performed. A curve showing the dependence of the SPRi signal on the streptavidin concentration was plotted (Figure 4). To avoid surface overload and steric hindrance, the appropriate streptavidin concentration was 0.5 mg/mL, just before the plateau appeared. At the same time, while testing for the appropriate streptavidin concentration, the appropriate assay buffer solution was tested. Two solutions were tested: HEPES-EDTA-P20 and PBS-EDTA-P20. The plotted curves are shown in Figure 4. The buffer composition selected for the study was HEPES-EDTA-P20.

### 3.2. Saturation of the Biosensor Surface with Antibodies: Selection of the Appropriate Ligand Concentration

**Figure 5:**
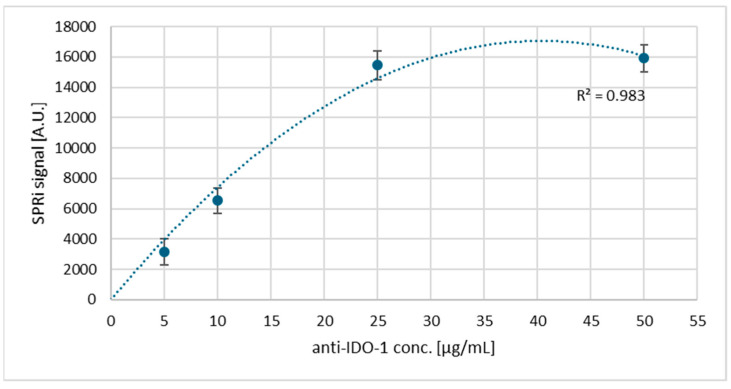
Dependence of the SPRi signal on the IDO-1 antibody concentration.

**Figure 6:**
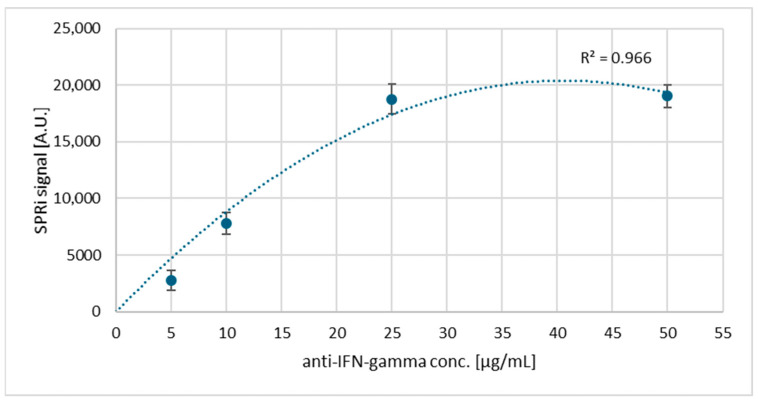
Dependence of the SPRi signal on the IFN-γ antibody concentration.

Antibody solutions with concentrations from 5 μg/mL to 50 μg/mL were tested, and curves were drawn showing the dependence of the SPRi signal on the antibody, separately, for IDO-1 and IFN-γ (Figure 5 and Figure 6). Each measurement was performed 3 times, from which the standard deviation was then calculated, constituting the graph’s error bars. The SPRi signal measurement was studied after immobilizing IDO-1 and IFN-γ-specific antibodies on the streptavidin surface. For both analyses, the concentration chosen was the one that appeared at the beginning of the plateau in the saturation curves, i.e., 25 μg/mL.

### 3.3. Selection of Rinse Frequency

**Figure A1:**
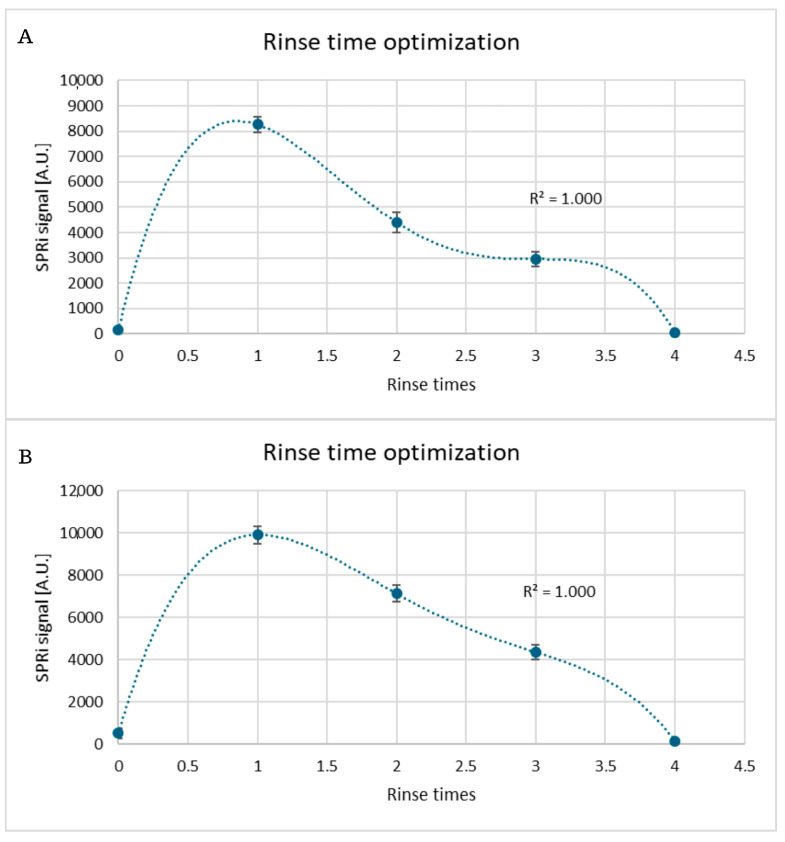
Dependence of the SPRi signal on the number of washes for (**A**) IDO-1 and (**B**) IFN-γ. In both cases, the optimal value of washes is a single wash with the basic buffer. With each subsequent wash, a decrease in the SPRi signal is observed on the graph. This may indicate an undesirable dissociation of the analyte molecules bound to the receptor surface.

Rinsing the biosensor surface is an essential step prior to measurement, as it removes unbound particles originating from the sample matrix. The presence of these non-specifically adsorbed components can interfere with the measurement by increasing the energy required to excite surface plasmons, thereby reducing the sensitivity and accuracy of the analysis. Supplying more energy is often impossible due to instrument limitations. To select the appropriate number of washes, standard solutions of IDO-1 and IFN-γ at a concentration of 10 ng/mL were prepared and applied to the biosensor surface. Excess protein was aspirated after a fixed reaction time (10 min), and washing was performed. The first three washes were performed with the basic buffer (HEPES–EDTA–P20) and the last wash was performed with distilled water. Data acquisition was performed after each wash. The figures on the dependence of the SPRi signal on the number of washes for IDO and IFN-γ are featured in Appendix A (Figure A1).

### 3.4. Proof of Layer Formation on the Sensor Surface Using SPR Curves

**Figure 7:**
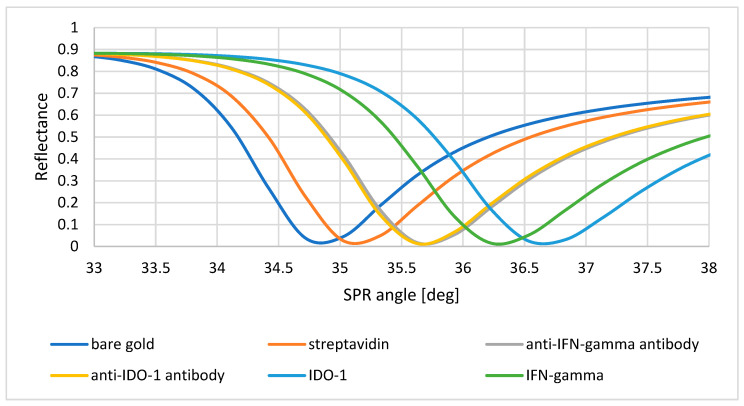
SPR curves for the layers forming the biosensor within the angular range from 33 to 38 degrees.

To prove that the formation of layers on the biosensor surface had taken place, SPR curves were plotted. They show the dependence of the change in reflectance as a function of the angle of refraction. Reactions were carried out to enable the formation of successive layers on the surface, and data were acquired for each layer at angles from 33 to 38 degrees. In Figure 7, we can observe the shifting of each layer towards a higher angle than the preceding layer. This enabled us to confirm that the formation of layers on the biosensor surface had occurred. The SPR curves for the IDO-1 and IFN-γ-specific antibodies overlap, due to the similar masses of both antibodies.

### 3.5. Association Time and Regeneration Cycles

**Figure A2:**
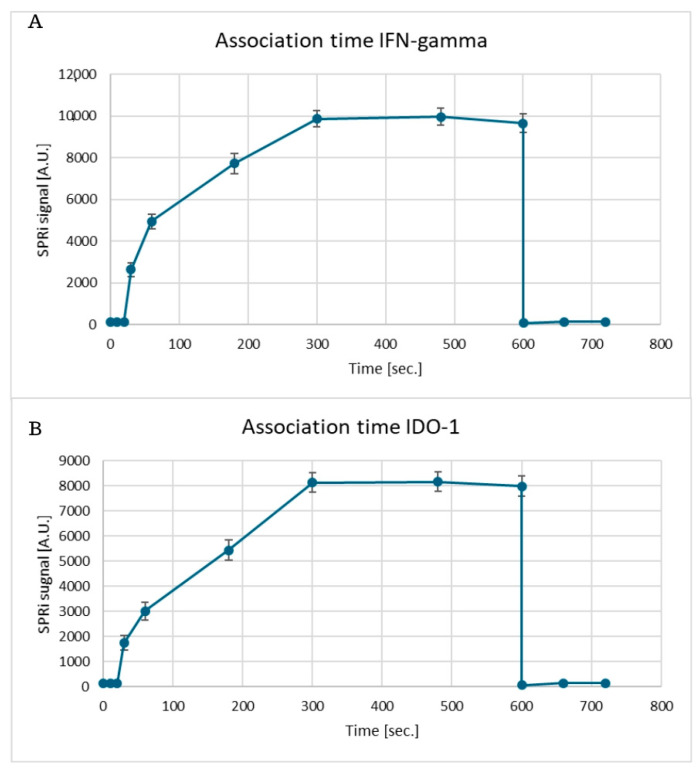
SPRi signal vs. reaction time curves for (**A**) IDO-1 and (**B**) IFN-γ samples; the concentration of standard protein solutions is 10 ng/mL, the concentration of receptor layers is 25 μg/mL. The SPRi signal values in both cases stabilize at about 5 min. This means that the optimal reaction time for both proteins is 5 min. The figures also show the regeneration process, i.e., NaOH removal of the analyte and receptor layer from the sensor surface. After regeneration, the generated signal returns to the baseline.

The interaction time between the receptor and the protein varies depending on the method used. To investigate the optimal time according to which the reaction occurs, the analyte was applied to the receptor layer at a concentration of 25 μg/mL; data acquisition was performed and standard solutions of IDO-1 and IFN-γ proteins at concentrations of 10 ng/mL were also applied to examine the association time, and then the dependence curves of the SPRi signal on the reaction time were developed. The dependence curves are featured in Appendix A*—*Figure A2. The graphs indicate that the SPRi signal values in both cases stabilize at about 5 min, which is the optimal time for the antibody to react with IDO-1 and IFN-γ. The biosensors are regenerated to reduce the analysis time and the amount of materials used. This can adversely affect the accuracy of the measurements. Standard solutions of IDO-1 and IFN-γ proteins were prepared and applied to the biosensor surface, and measurements were performed to assess the optimal number of regenerations. Then, the surface was regenerated using a 50 mM NaOH solution, which was applied to the biosensor surface for 3 min, then removed and the surface was washed with distilled water. A graph of the presentation of the experiment is provided in Appendix A—Figure A3 and Figure A4. The graphs show the effect of the number of regeneration cycles on the results, and the assumed relative error limit is 10%. In the case of the IDO-1 assay, the measurement accuracy decreases after the 6th regeneration cycle, while in the case of IFN-γ, the accuracy decreases significantly after the 7th regeneration cycle.

## 4. Validation of the Analytical Method

### 4.1. Analytical Response of the Biosensor, Limit of Detection and Quantification

**Figure 8:**
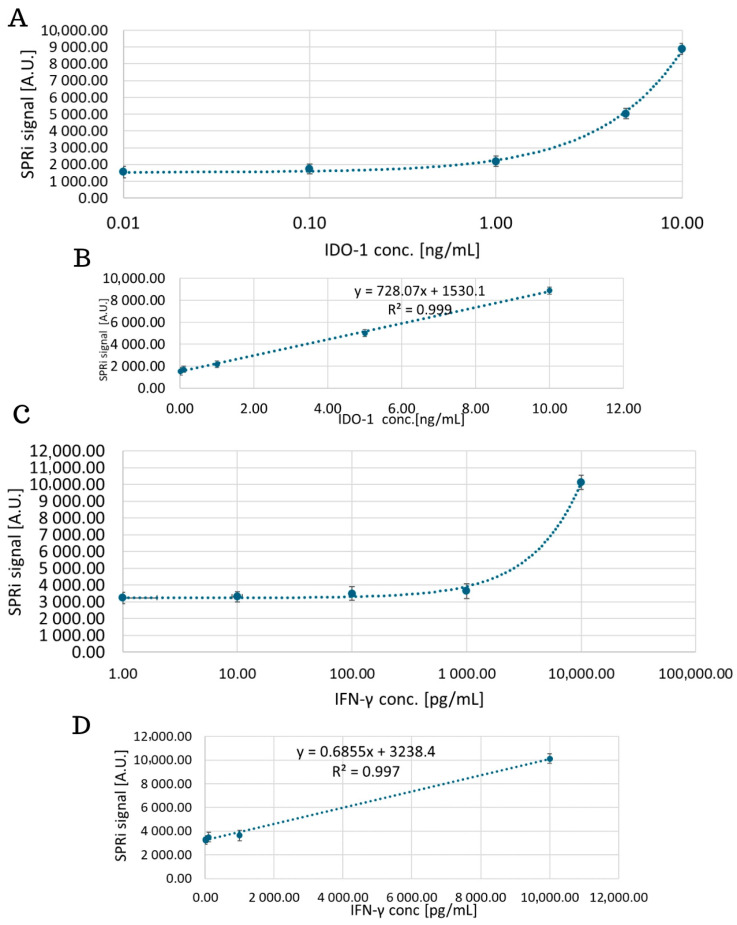
Dependence of the SPRi signal on the concentration of the tested protein IDO-1 (**A**,**B**) and IFN-γ (**C**,**D**). The figure shows a linear relationship with a logarithmic X-axis scale. The logarithmic scale (**A**,**C**) was used only to improve the readability of the graph. The concentration of the receptor layer for both proteins was 25 μg/mL.

The analytical response of the biosensor was examined by analyzing the SPRi signal of the standard solutions in the concentration range for IDO-1 from 0.01 ng/mL to 10 ng/mL and for IFN-γ from 0.1 pg/mL to 1000 pg/mL. The obtained curves are shown in Figure 8. The equations of the curves were also determined, which then enabled the concentrations of the tested proteins in the biological samples to be determined. In the next stage, the detection and quantification limits were determined. These parameters were calculated based on the standard deviation of the signals obtained for the blank sample, namely the HEPES–EDTA–P20 solution. The results are presented in Table 1. They were calculated using the following formulas: LOD = 3 × SD_BLIND TEST_; LOQ = 10 × SD_BLIND TEST_; where SD is the standard deviation of the results obtained for the blank sample.

### 4.2. Precision and Accuracy

Evaluation of the precision and accuracy of the developed method enables an assessment of both the consistency between the repeated measurements and the agreement between the measured concentration and the true analyte concentration. Standard solutions of proteins with different concentrations were prepared, which are listed in Table 2. Each measurement was performed 3 times. The mean concentration, standard deviation, the parameter defining precision, namely the coefficient of variation (CV), and the parameter of accuracy, namely the relative error, were determined. Low CV parameters and a low relative error indicate the high precision and accuracy of the developed method. The following formulas were used: %error = ((C_mean_ − C_real_)/C_real_) × 100 and CV = (C_mean_/SD) × 100; where C_mean_ is the mean protein concentration obtained during the measurement, C_real_ is the actual protein concentration, CV is the coefficient of variation [%], and SD is the standard deviation of the obtained results.

### 4.3. Repeatability and Selectivity of the Method

Repeatability is a specific indicator of the usefulness of the developed method. It enables the consistency of the results with the same parameters to be assessed. The biosensor for determining IDO-1 and IFN-γ must be adapted to carry out determinations using various biological materials, with a focus on maintaining the high repeatability of the results. In order to assess the repeatability of the method, a 5-fold test was performed on a real sample, namely blood serum and tissue homogenate from patients diagnosed with brain glioma, and the tested proteins were determined. The samples were diluted 5-fold for IFN-γ and 2-fold for IDO-1 in serum, while the tissue homogenate samples were diluted 2-fold for IFN-γ and IDO-1. The results are presented in Table 3.

The presence of components from the biological sample matrix may interfere with the analytical signal, which causes the generation of erroneous analysis results. To check whether interferents affected the determination of IDO-1 and IFN-γ, a study was performed using proteins at concentrations of 5 ng/mL, namely IDO-1, IFN-γ, VEGF-A, VEGF-R2, FGF-2, NRP-1, cathepsin B, and cathepsin S. The proteins selected to determine the selectivity of the method occur in the brain and belong to proinflammatory proteins. Each measurement was repeated 3 times, and then the average SPRi signal value and the concentration value generated by the interferents were determined. The results are presented in Table A1 in Appendix A.

### 4.4. Measurement in Biological Material

To check the performance of the biosensor, IDO-1 and IFN-γ were simultaneously determined in biological samples, namely plasma and urine from patients diagnosed with bladder cancer, as well as in the plasma and urine from patients in the control group who were diagnosed with prostatitis. The samples were appropriately diluted so that the concentrations were within the linear concentration range of the biosensor, as follows:

(A) plasma samples were diluted 5-fold for IFN-γ determinations and 2-fold for IDO-1;
(B) urine samples were diluted 5-fold for IFN-γ determinations and 1-fold for IDO-1.

The measuring chip contained receptor layers for IDO-1 and IFN-γ determinations, which enabled us to determine these biomarkers simultaneously during a single measurement cycle. The results of the urine and plasma concentrations of the patients with bladder cancer and those from the control group are presented in Table A2 and Table A3 in Appendix A. To confirm the correctness of the performed tests, the results were also compared to the values obtained using a certified method, namely an ELISA test using the same plasma and urine samples.

#### Analysis of the Obtained Results Using the SPRi and ELISA Method

**Figure 9:**
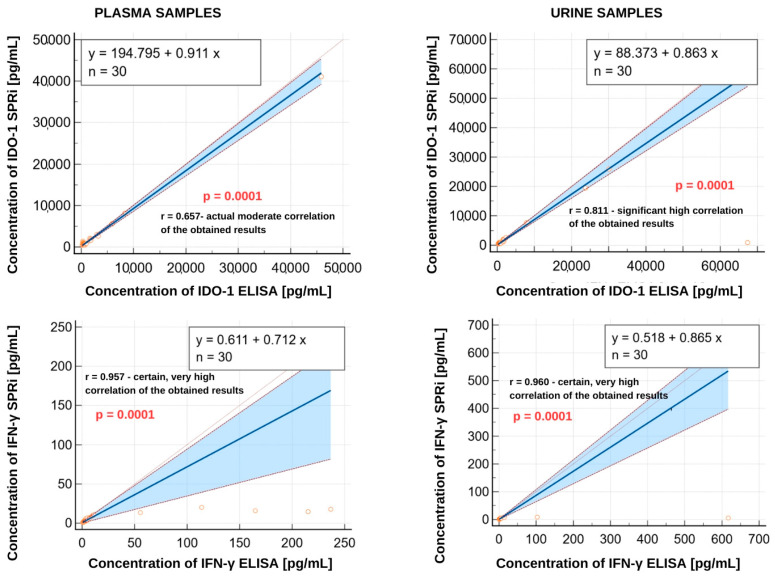
Passing–Bablok test results for the plasma and urine samples from patients diagnosed with bladder cancer and the controls obtained by ELISA (reference method) and SPRi (candidate method).

The agreement between the two measurement methods was compared using the Passing–Bablok test. The *p* parameters and the values of the regression coefficients r were determined and then interpreted according to the J. Guilford scale. The obtained analysis graphs are presented in Figure 9. According to the obtained results, all the results are statistically significant (*p* = 0.0001). The correlation coefficient values indicate that the results obtained from the two methods, the reference ELISA and the candidate SPRi method, are convergent.

## 5. Results and Discussion

The first stage of the experiments performed included optimizing the method conditions. Selecting the appropriate streptavidin concentration to be applied on the measuring plate enables the maximum concentration that needs to remain on the chip without unnecessary surface washing to be determined. In Figure 4, the most suitable concentration of streptavidin corresponds to the value of 0.5 mg/mL, according to which the SPRi signal increases to this value, then stabilizes; we observe this as a plateau on the saturation curve. Simultaneous testing of the measurement buffer indicates that the most suitable test buffer is HEPES–EDTA–P20, which facilitates the formation of an amide bond and binds streptavidin to biotin. The PBS–EDTA–P20 buffer solution causes difficulties in binding biotin to streptavidin.

The next important step is to select the appropriate antibody concentration that will form the receptor layer of the dextran biosensor. Here, curves are plotted, according to which we observe an increasing SPRi signal up to a specific concentration value (25 μg/mL), which then decreases and stabilizes. The antibody no longer binds above a 25 μg/mL concentration, so it was selected as the optimal value. Another important aspect during the construction of the biosensor is the selection of the number of washes required. This enables the removal of unbound biological material and excess reagents that may interfere with the sensor’s operation. In addition, too many washes may worsen the repeatability between samples. Appendix A.1 indicates that in both cases, the optimal value of washing is a single wash with the basic buffer. With each subsequent wash, we observe a decrease in the SPRi signal on the graph. This may indicate the undesirable dissociation of the analyte molecules bound to the receptor surface. The estimated K_D_ values for the antibodies of both proteins indicate medium-to-high affinity (K_D_ < 10^−^^7^), which meets the requirements for applications in regard to the receptor layer. In addition, the next part of the experiment, i.e., the confirmation of the presence of layers formed on the gold surface, perfectly illustrates the situation on a thin metal layer, namely shifts of the curves towards higher angle values (Figure 7) indicate the presence of antibody layers in both cases and, subsequently, the presence of a layer of immobilized IDO-1 and IFN-γ proteins, which confirms good affinity and the lack of antibody dissociation, despite only a single wash taking place.

**Figure A3:**
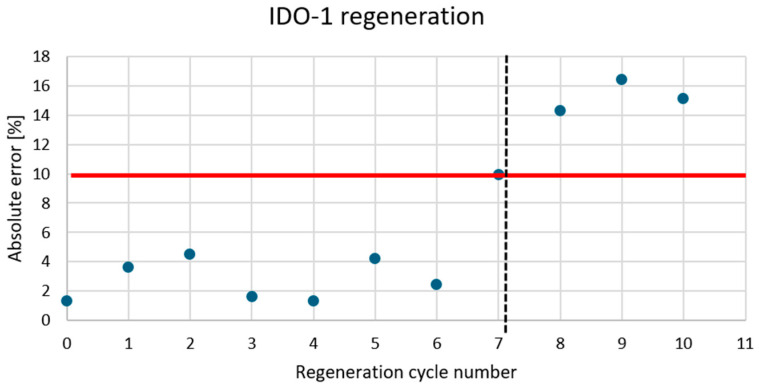
The influence of regeneration on the accuracy of the obtained IDO-1 measurement results.

**Figure A4:**
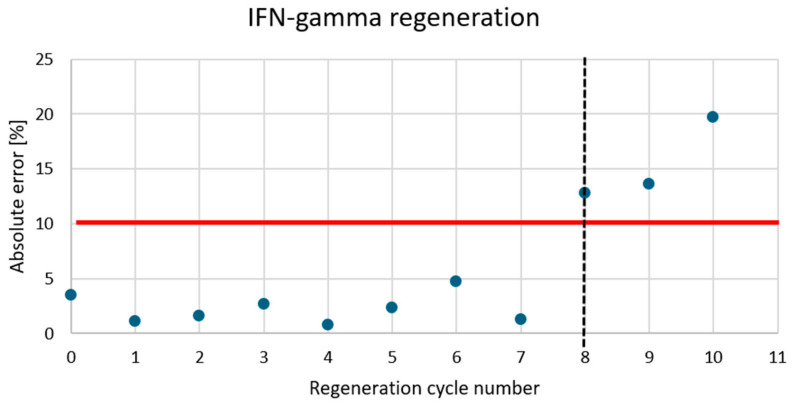
The influence of regeneration on the accuracy of the obtained IFN-γ measurement results.

The association time indicates how quickly the analyte binds after applying the sample to the receptor layer. This enables the affinity of the molecules to be estimated, the optimal measurement time in the biosensor to be determined, and the selection of the appropriate receptor. Figure A2 in Appendix A shows that the optimal reaction time is 5 min; at the same time, the regeneration shown in the graph indicates that nothing remains on the sensor surface that causes a residual signal, namely that it returns to the baseline. According to the studies carried out, regeneration can be performed up to seven times, corresponding to returns to the baseline (Figure A3, Appendix A).

Validation of the method enabled the dynamic ranges for IDO-1 from 0.01 ng/mL to 10 ng/mL and for IFN-γ from 0.1 pg/mL to 1000 pg/mL to be determined, which enabled the limits of detection and quantification to be determined (Table 1). For IDO-1, the LOD was obtained at 0.27 pg/mL and LOQ = 0.81 pg/mL. For IFN-γ, LOD = 1.76 pg/mL and LOQ = 5.29 pg/mL. The level of IDO-1 is determined mainly by its activity. The concentration of IDO-1 in serum was determined by Chen et al. and was on average 25.90 ± 4.91 ng/mL [22] and also by Bao et al. [21] and was obtained on average at 8.57 ± 3.19 ng/mL for healthy volunteers. In healthy individuals, according to [40], levels of IFN-γ in serum are 18.8 ± 3.89 pg/mL, and according to [41], 17.33 pg/mL. Some studies have reported higher values, such as 136 pg/mL in blood serum of healthy individuals [27].

The calculated precision and accuracy parameters at a very low level indicate that the results obtained using this method are characterized by high precision and accuracy. A low SD and CV also indicate repeatable results (Table 3). We also know that in regard to interfering substances, interferents do not directly affect the analysis results. The obtained concentrations of interfering substances are below the detection limits of IDO-1 and IFN-γ. The source of the signal is, therefore, undoubtedly the antibody–protein reaction.

The biosensor was used to test for IDO-1 and IFN-γ in the plasma and urine samples from patients diagnosed with bladder cancer and from patients in the control group. The obtained results are presented in Appendix A, Table A2 and Table A3. The tables also contain the results obtained using a certified laboratory method, an ELISA. The Passing–Bablok analysis used the comparative test as a nonparametric regression method. It is used to assess the agreement between the reference and candidate methods. This test enables the detection of both systematic and proportional errors. The graphs in Figure 9 show pairs of measurements, x as the reference method and y as the candidate method, and the Passing–Bablok regression line, the line of perfect agreement y = x. For IDO-1 determinations in plasma, a moderate correlation was obtained between the reference and candidate methods. The r coefficient (0.657) indicates high variability of the measurements, according to which there is a probability of random error or systematic differences. The confidence interval (CI) range suggests that the association’s strength is unstable (0.389–0.823). In regard to the urine samples, however, there is a high correlation between the methods. The confidence interval is relatively narrow (0.636–0.906), indicating a stable and strong linear association. The data suggest that the candidate method for urinary IDO-1 determinations is reliable.

A very high correlation was observed for IFN-γ in both the plasma and urine samples, indicating an almost complete concordance between the results obtained using the two analytical methods. High r values (in plasma 0.957 and urine 0.960) and narrow confidence intervals (0.910–0.979 in plasma, 0.918–0.981 in urine) suggest that the candidate method perfectly reproduces the reference method. The statistical significance of *p* = 0.0001 in all cases means that the correlation is not coincidental. The lowest correlation was obtained for IDO-1 in plasma. This may be due to the low stability of IDO-1 in plasma; it is less stable outside the cell [42]. The studied population was clinically diverse, including patients with different stages of disease; thus, the expression and release of IDO-1 may be variable, which additionally affects the correlation [43].

## 6. Conclusions

The constructed SPRi biosensor for IDO-1 and IFN-γ may have applications in regard to diagnosing bladder cancer using both urine and plasma samples from patients with this disease. However, the diagnostic utility of these potential biomarkers needs to be tested in larger numbers of samples and more diverse patient groups.

## Figures and Tables

**Table 1:** Detection and quantification limits of the method.

Analyte	LOD	LOQ
IDO-1	0.27 ng/mL (0.1 ng/mL [22] 0.278 ng/mL [25])	0.81 ng/mL
IFN-γ	1.76 pg/mL (5.7 pg/mL [27] 17 pg/mL [28])	5.29 pg/mL

**Table 2:** Precision and accuracy parameters for the IDO-1 and INF-γ determination methods.

Precision and Accuracy					
IDO-1	Concentration [ng/mL]				
	0.81	1.00	5.00	10.00	
I measurement	0.87	1.01	5.03	9.89	
II measurement	0.94	0.89	4.93	9.94	
III measurement	0.83	0.98	5.01	9.96	
Mean concentration [ng/mL]	0.88	0.98	4.99	9.93	
SD [ng/mL]	0.06	0.06	0.05	0.04	Mean
CV [%]	6.33	6.51	1.06	0.36	3.56
%error	8.64	4.00	0.20	0.70	3.39
IFN-γ	Concentration [pg/mL]				
	5.29	10.00	1000.00	10,000.00	
I measurement	5.78	9.92	996.76	9998.64	
II measurement	5.69	10.46	1002.45	9985.35	
III measurement	5.34	9.79	993.32	9997.75	
Mean concentration [pg/mL]	5.60	10.06	997.51	9993.91	
SD [pg/mL]	0.23	0.36	4.61	7.43	Mean
CV [%]	4.15	3.53	0.46	0.07	2.05
%error	5.92	0.57	0.25	0.06	1.70

**Table 3:** Parameters concerning the repeatability of the method for determining IDO-1 and IFN-γ in serum samples and homogenates.

Blood Serum			
IDO-1	Concentration [ng/mL]	IFN-γ	Concentration [pg/mL]
I	38.28	I	190.1
II	35.97	II	188.76
III	37.64	III	195.03
IV	39.76	IV	183.54
V	37.55	V	197.37
Mean concentration [ng/mL]	37.84	Mean concentration [pg/mL]	190.96
SD [ng/mL]	1.37	SD [pg/mL]	5.44
CV [%]	3.62	CV [%]	2.85
Tissue homogenate			
IDO-1	Concentration [ng/mL]	IFN-γ	Concentration [pg/mL]
I	22.66	I	2.4
II	21.75	II	2.23
III	23.02	III	2.51
IV	22.89	IV	2.42
V	20.93	V	2.32
Mean concentration [ng/mL]	22.25	Mean concentration [pg/mL]	2.38
SD [ng/mL]	0.89	SD [pg/mL]	0.11
CV [%]	4.00	CV [%]	4.46

## Data Availability

Dataset available on request from the authors.

## Appendix A

### Appendix A.5

**Table A1:** Selectivity of the IDO-1 and IFN-γ determination method.

Selectivity of the Method							
IDO-1 (antibody)		SPRi signal			Mean	Concentration [ng/mL]	<LOD
		I	II	III			
IFN-γ	5 ng/mL	1539.71	1531.35	1544.79	1538.62	0.01	
VEGF-A		1541.59	1536.93	1556.67	1545.06	0.02	
VEGF-R2		1537.2	1545.28	1540.48	1540.99	0.02	
FGF-2		1539.88	1538.97	1537.41	1538.75	0.01	
NRP-1		1531.05	1544.03	1547.76	1540.95	0.02	
KAT B		1544.18	1531.47	1534.76	1536.80	0.01	
KAT S		1546.58	1545.14	1535.12	1542.28	0.02	
IFN-γ (antibody)		SPRi signal			Mean	Concentration [pg/mL]	<LOD
		I	II	III			
IDO-1	5 ng/mL	3240.09	3240.19	3238.08	3239.45	1.54	
VEGF-A		3236.85	3240.61	3239.24	323,890	0.73	
VEGF-R2		3240.66	3238.8	3239.12	3239.53	1.64	
FGF-2		3239.65	3240.11	3237.06	3238.94	0.79	
NRP-1		3240.68	3237.89	3237.38	3238.65	0.36	
KAT B		3238.01	3241.37	3236.94	3238.77	0.54	
KAT S		3235.86	3240.96	3240.63	3239.15	1.09	

### Appendix A.6

**Table A2:** Concentrations of IDO-1 and IFN-γ obtained in the assay of these proteins in the blood plasma of patients with bladder cancer and patients in the control group using the constructed SPRi biosensor, as well as a certified method, the ELISA test.

Plasma Sample Number	Concentration of IDO-1 [pg/mL]	Concentration of IDO-1 ELISA Test [pg/mL]	Concentration of IFN-γ [pg/mL]	Concentration of IFN-γ ELISA Test [pg/mL]
1	1509.72	1520	19.85	113.7
2	456.70	200	2.20	1.63
3	1628.80	1830	1.00	0.54
4	689.50	560	15.72	165
5	679.99	730	3.59	4.88
6	662.44	710	5.12	4.34
7	2070.24	1663	9.64	10.21
8	1891.92	1800	2.50	2.17
9	732.97	490	7.46	7.05
10	663.85	320	3.04	3.28
11	502.35	440	3.12	3.79
12	3013.72	2980	14.55	215.2
13	41,110.98	45,870	6.66	4.93
14	5616.21	5890	3.49	4.31
15	8132.62	8330	17.65	236.8
16	556.04	380	2.15	2.17
17	882.31	830	9.76	9.76
18	870.71	570	6.52	7.59
19	538.88	590	5.64	3.31
20	539.17	580	0.99	1.17
21	681.42	600	3.51	2.17
22	857.27	470	10.78	11.11
23	877.36	950	2.41	3.22
24	665.94	460	3.24	2.58
25	720.20	670	1.85	2.17
26	950.90	320	1.51	1.31
27	457.22	640	0.91	0.59
1C	1419.58	320	0.94	1.08
2C	1070.46	330	13.61	55.34
3C	2591.03	3220	3.81	2.71

### Appendix A.7

**Table A3:** Concentrations of IDO-1 and IFN-γ obtained in the assay of these proteins in the urine samples of patients with bladder cancer and patients in the control group using the constructed SPRi biosensor, as well as a certified method, the ELISA test.

Urine Sample Number	Concentration of IDO-1 [pg/mL]	Concentration of IDO-1 ELISA Test [pg/mL]	Concentration of IFN-γ [pg/mL]	Concentration of IFN-γ ELISA Test [pg/mL]
1	19,510.56	23,750	1.99	1.63
2	25,945.57	30,060	1.72	1.14
3	7502.61	8290	3.27	3.21
4	919.71	860	1.79	1.17
5	814.21	640	2.96	3.25
6	1344.73	1750	0.78	1.08
7	2048.75	1640	0.51	0.54
8	2586.19	2370	1.84	1.08
9	642.32	950	0.94	1.17
10	1005.24	630	0.83	0.93
11	560.36	580	2.90	2.12
12	647.91	780	3.48	3.18
13	452.52	310	3.21	3.13
14	615.57	440	3.26	2.91
15	1122.50	1530	3.35	3.27
16	493.11	600	8.35	103.2
17	1954.25	1840	8.85	12.17
18	689.10	780	6.99	14.27
19	728.06	800	5.72	4.19
20	621.15	670	5.55	5.14
21	831.24	67,350	4.26	3.29
22	1853.03	1690	2.89	2.17
23	1308.13	1480	5.71	5.11
24	999.90	650	3.33	3.22
25	7620.39	7810	4.30	4.36
26	772.79	730	2.37	2.17
1C	686.73	660	5.62	616.8
2C	500.58	260	1.45	1.08
3C	613.92	840	0.72	0.54
4C	645.75	750	4.22	6.23
